# Microalgae-enriched (bio)inks for 3D bioprinting of cultured seafood

**DOI:** 10.1038/s41538-025-00386-y

**Published:** 2025-02-12

**Authors:** Diana M. C. Marques, Madalena Jabouille, Afonso Gusmão, Marco Leite, Paola Sanjuan-Alberte, Frederico Castelo Ferreira

**Affiliations:** 1 Department of Bioengineering and Institute for Bioengineering and Biosciences, Instituto Superior Técnico, Universidade de Lisboa, Lisbon, Portugal; 2 Associate Laboratory i4HB—Institute for Health and Bioeconomy, Instituto Superior Técnico, Universidade de Lisboa, Lisbon, Portugal; 3 IDMEC, Instituto Superior Técnico, Universidade de Lisboa, Lisbon, Portugal

**Keywords:** Biomaterials, Environmental biotechnology

## Abstract

Cultured seafood offers a sustainable alternative to traditional seafood by eliminating the need for animal sacrifice and reducing environmental impacts. 3D bioprinting enables precise manufacturing of these products by combining animal cells with plant-based materials. This study introduces novel (bio)inks: (i) κ-CAM bioinks (κ-carrageenan, alginate, and methylcellulose) compatible with seabass cells; and (ii) mFAT inks, plant-based fat inks containing microalgae for enhanced organoleptic properties. κ-CAM bioinks revealed Young’s modulus between 14.62 and 25.70 kPa values, suitable for cultured seafood products. Both κ-CAM and mFAT formulations presented adequate printability (Pr~1). *Dicentrarchus labrax* Embryonic Cells, encapsulated in κ-CAM bioinks, maintained viabilities >76.14% for up to 15 days. A preliminary assessment confirmed that specific microalgae can enhance the sea-like smell and flavor of the mFAT ink, and a 3D-printed calamari was fabricated to showcase its potential in the manufacturing of complex structures. Finally, hybrid structures combining both types of (bio)inks were also developed.

## Introduction

Environmental concerns related to fishing and intensive fish farming practices—such as habitat destruction and water pollution caused by antibiotics^1,2^ and microplastics^3^—highlight the need for more sustainable seafood production strategies. Moreover, with the global human population projected to reach 9.7 billion people by 2050^4^, a significant increase in food demand is expected, further exacerbating the environmental burden of food systems^5^.

The development of cultured fish products offers an alternative solution for food supply, with the potential to protect the planet’s ecosystems, promote sustainability, and improve animal welfare^6^. The growing interest in the cultured seafood field has led to the launch of more than 54 companies focused on these products to date^7^.

Even though plant-based products can resemble the appearance of animal-derived food products, reports indicate that they still require improvements in sensorial properties such as taste and smell, and nutritional value^8,9^. This is also critical for cell-based products which should have the adequate sensorial properties to be accepted by consumers^10^.

Different sources of microalgae have been previously explored to enhance the organoleptic properties of plant-based fish^11^, however, their application as nutritional and sensorial enhancers in scaffolds for cell-based cultured fish has not yet been reported. Microalgae are a rich source of beneficial lipids, including the omega-3 long-chain polyunsaturated fatty acids (PUFAs) such as eicosapentaenoic acid (EPA) and docosahexaenoic acid (DHA), which are key components of the fatty acid composition of fish^11^. Although omega-3 PUFAs are commonly associated with fish meat, fish themselves are not primary producers of PUFAs. Microalgae are responsible for producing more than half of the total EPA and DHA in the biosphere, which accumulate in fish through the food chain^12^. *Nannochloropsis oceanica* (NO)*, Tetraselmis chuii* (TC), and *Phaeodactylum tricornutum* (PT) are examples of microalgae’s with capacity to produce high amounts of EPA and DHA^13,14^. Besides this feature, these microalgae are also promising flavoring agents for alternative seafood, with high umami taste and active seafood odor^15^.

Furthermore, 3D extrusion (bio)printing can contribute to enhancing the texture of cultured fish by producing complex structures that replicate key meat-like features^16–18^. However, many materials used in the formulation of bioinks often derive from animal sources, such as gelatine, undermining the animal ethical goal associated with cultured meat and seafood production^19^.

Alginate, the most commonly used material in algae-based bioink formulations, often requires the use of the freeform reversible embedding of suspended hydrogels (FRESH) method. This method involves extruding low viscosity directly into a sacrificial gelatine support bath, which still relies on animal-derived materials^20^. In addition, the retrieval of FRESH 3D-printed structures involves melting the gelatine support bath at 37 °C, a temperature suitable for mammalian cells but potentially compromising the biocompatibility of fish cells, typically cultured at lower temperatures. Alternative support materials such as agar or agarose, require mechanical removal of the printed structures, which can potentially cause irreversible damage. Nonetheless, alginate extrusion bioprinting has also been reported without the need for FRESH. In these studies, alginate is typically combined with gelatine and collagen or subjected to physical or chemical modifications. Strategies to enhance alginate strength during extrusion bioprinting include crosslinking through ionic interactions with divalent cations (e.g., Ca^2+^, Sr^2+^, and Ba^2+^) or photo-crosslinking^21^.

Herein, we propose a strategy to 3D bioprint self-standing, cell-based structures composed of plant-based materials supplemented with microalgae and omega-3, designed to be compatible with fish cells and aimed at manufacturing cultured seafood products. To achieve this, bioinks consisting of κ-carrageenan, alginic acid and methylcellulose (κ-CAM) were formulated to support living seabass fish cells. κ-CAM bioinks were characterized and tuned to be bioprinted at temperatures compatible with fish cultures, ranging from 20 °C to 28 °C. A second ink consisting of plant-based oils, omega-3 and three different microalgae (NO, TC, and PT) was developed to formulate plant-based microalgae-enriched material (mFAT). By combining these components, we ensure that the κ-CAM bioink formulations contain gelation agents (carrageenan and alginate), a thickening agent (methylcellulose), and an animal component (seabass cells), while the mFAT inks provide fat content (plant oils), omega-3 (algae oil), and sensorial enhancers (microalgae) to the final product. Once the printability of both materials was established, a hybrid cultured fish product was manufactured combining both κ-CAM and mFAT (bio)inks. To the best of our knowledge, this study presents for the first time a strategy for the 3D bioprinting of fish cells using exclusively plant and algae-based materials combined with fat structures that contain microalgae towards its organoleptic enhancement.

## Results

### Formulation and characterization of the κ-CAM bioinks

**Fig. 1:**
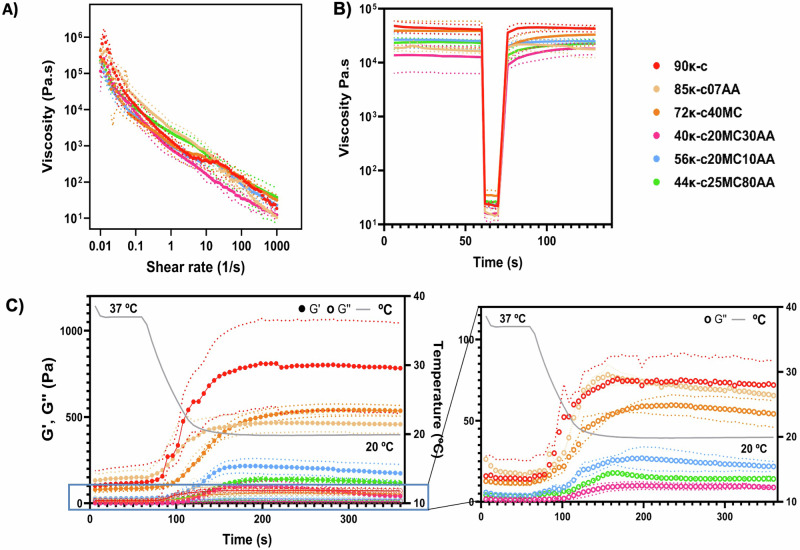
Rheological characterization of the κ-CAM bioinks. **A** Flow curves of the shear rate sweep test of the κ-CAM bioinks as a function of shear rate (0.01–1000 s^−1^), showing their shear-thinning behavior, indicated by the decrease in viscosity with increased shear rates. Data is shown as mean ± SD (*N* = 3). **B** The recovery behaviors of the κ-CAM bioinks by applying an initial shear stress of 2 Pa for 60 s, followed by 15 Pa during 30 s, and a final shear stress of 2 Pa for 60 s. Data is shown as mean ± SD (*N* = 3). **C** The left graph shows the gelation kinetics of the κ-CAM bioinks showing the storage (G′, filled circles) and loss moduli (G″, non-filled circles) throughout a temperature sweep from 37 °C to 20 °C, over 360 s. The right graph outlines the values between 0 Pa and 100 Pa, respective to the G″. Solid lines indicate the mean values and dotted lines indicate the standard deviation. Data is shown as mean ± SD (*N* = 3). A specific color was allocated for the data related to each ink, as labeled (top right corner).

For the development of bioinks compatible with seabass fish cells, several formulations containing different concentrations and ratios of κ-carrageenan (κ-c), alginic acid (AA) and methylcellulose (MC) were initially prepared (see “Methods” for further details in their composition and denomination) and generically referred to as κ-CAM bioinks. Initially, rheological characterization was performed. Figure 1A shows a continuous decrease in viscosity with increasing shear rates across all κ-CAM bioinks, consistent with previous reports on κ-c, alginate^22^, and MC-containing bioinks^23^. This behavior indicates that all κ-CAM bioinks exhibit shear-thinning properties, an important characteristic for bioinks. Thus, demonstrating the potential to 3D print a layer-by-layer structure capable of maintaining its integrity for all κ-CAM bioinks.

The recovery time of κ-CAM bioinks was measured through three distinct phases with different pressures: (i) pre-printing state (2 Pa); (ii) nozzle state (15 Pa); and (iii) post-printing state (2 Pa). The results of this can be seen in Fig. 1B. All κ-CAM bioinks recovered fully, except for 72κ-c40MC which had a 14.36% viscosity loss. This indicates, that after bioprinting, the structures made of 72κ-c40MC may need to be submitted to a high degree of crosslinking to compensate for the loss of viscosity and maintain their integrity. Moreover, we observe that in 90κ-c, 85κ-c07AA, 56κ-c20MC10AA, and 40κ-c20MC30AA the recovery time was around 10 s while 44κ-c25MC80AA took around 20 s to recover its initial viscosity.

κ-c was selected as the most predominant element in all κ-CAM bioinks with the exception of the 44κ-c25MC80AA formulation. In order to assess its temperature-dependent molecular arrangement, the viscosity of the bioinks was evaluated throughout a range of temperatures (37–20 °C) (Fig. 1C). The experimentally obtained curves illustrate that the coil-to-helix transition occurs as the temperature reaches 33–23 °C, depending on the bioink composition. For the bioinks 90κ-c, 85κ-c07AA, 72κ-c40MC, and 56κ-c20MC10AA, the timeframe where gelation transition happens is longer, with viscosity increasing, during 90–120 s, between 33.42 and19.84 °C (Table S1).

### Mechanical properties of the κ-CAM bioinks

**Fig. 2:**
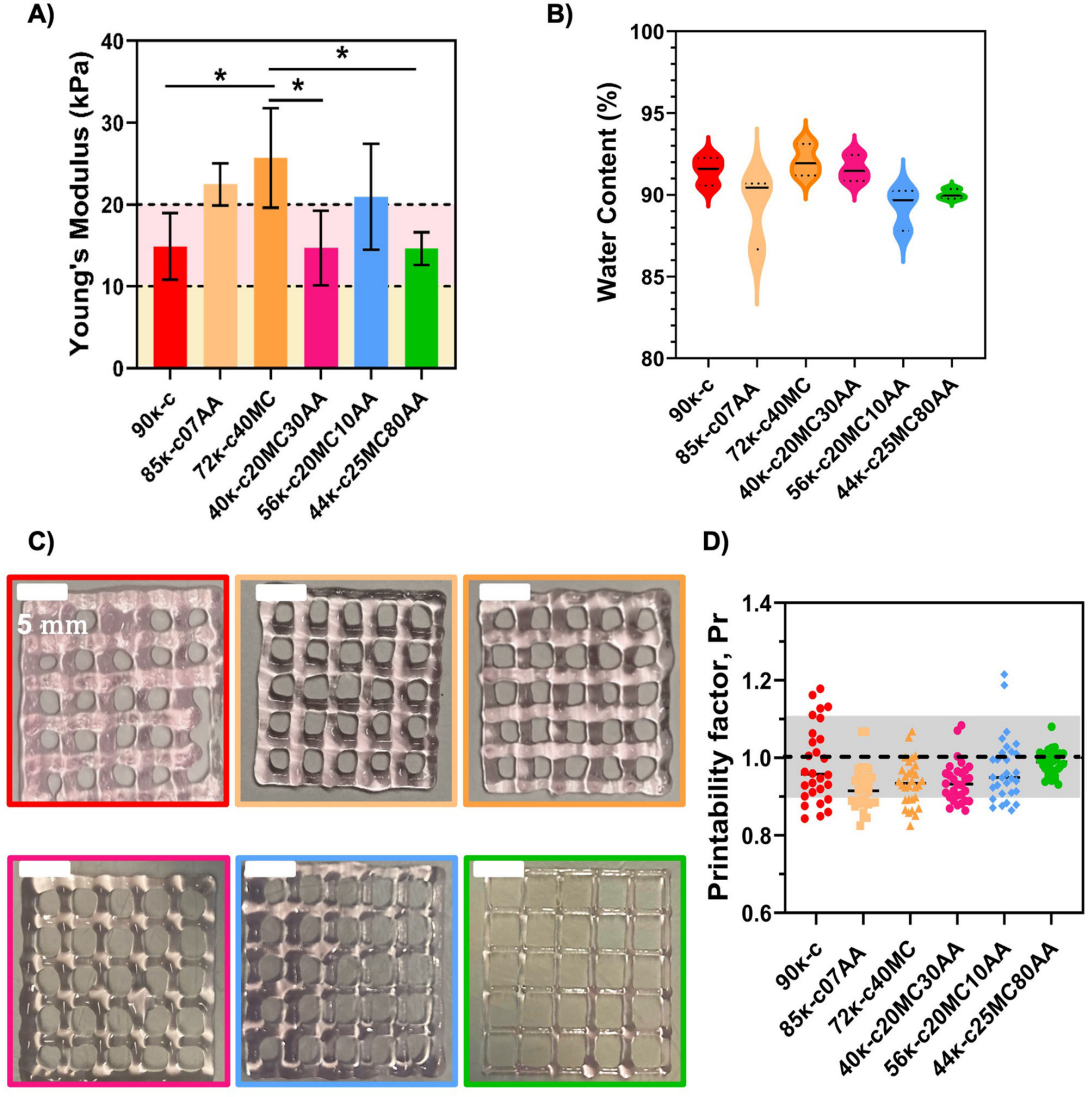
Mechanical properties and printability evaluation of κ-CAM bioinks. **A** Quantified Young’s modulus of hydrogels fabricated using the κ-CAM bioinks. Dotted lines at 10 kPa (light yellow area) and at 20 kPa (light pink area) represent the favorable Young’s modulus for fat cells and skeletal muscle cells, respectively. **B** Water content determination on all κ-CAM hydrogels. Statistical significance was assessed using ordinary one-way ANOVA and Tukey’s multiple comparison test (ns = *P* value ≥ 0.05; * = 0.01 <*P* value < 0.05; ** = 0.0016; *** = 0.0009; ****<0.0001). **C** Optical microscopy images of 3D-printed squared meshes using the κ-CAM bioinks. **D** Printability factors of the respective κ-CAM bioinks with the individual printability factor of each pore represented. The gray region marks the range of adequate ink printability (from 0.9 to 1.1) as described in Ouyang et al.^39^. Data are shown as mean ± SD (*n* = 3). Scale bar: 5 mm.

A uniaxial compression test was then performed on hydrogels cast with the κ-CAM bioinks, and the Young’s modulus was estimated from the linear region of stress–strain curves (Fig. S1). As shown in Fig. 2A, the obtained Young´s modulus ranged from 14.62 to 25.70 kPa. There are no significant statistical differences with the individual addition of AA and MC, except for the bioink 72κ-c40MC, which presents a significantly higher modulus. This might be due to the higher MC concentration present in this bioink, a component commonly used as a thickener^24^. Figure 2B shows the water content for the κ-CAM hydrogels. All scaffolds showed high water contents, above 89.24%, adequate values to support high cell viabilities^25^.

### Printability assessment of the κ-CAM bioinks

While the working temperature was already established, the printing speed, printing pressure, and needle inner diameter still required optimization. For this, different parameters were tested. The optimized printing values corresponded to 10 mm s^−1^ and 4–14.5 psi for printing speed and pressure, respectively. The selected needle’s inner diameter was 0.25 mm, except for 9ĸ-c which was 0.33 mm. A layer distance of 0.25 mm or 0.33 mm was maintained between printed layers, according to the diameter of the printing needle. Figure 2C shows 3D-printed 20 × 20 mm squared meshes. With these images, the printability was estimated by calculating the printability factor (Pr) from each pore of 3D printed meshes (Fig. 2D). This calculation determined that the printability of all κ-CAM bioinks fell within the acceptable printability region (Pr = 0.9–1.1).

### 3D bioprinting of seabass cells and viability evaluation

**Fig. 3:**
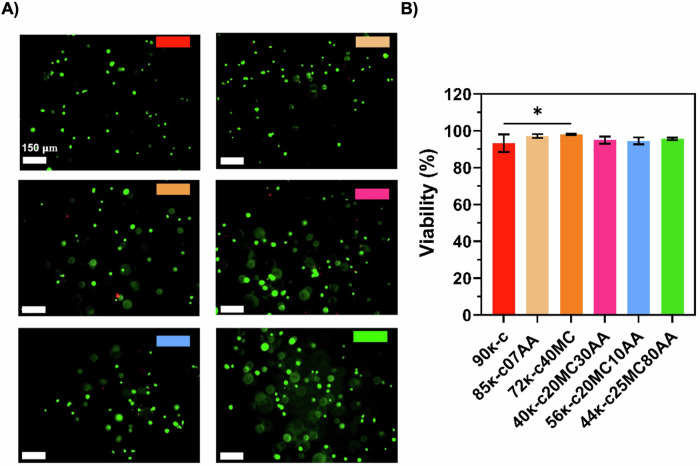
Evaluation of DLEC viability after bioprinting using κ-CAM bioinks. **A** Fluorescence microscope images of DLEC cells bioprinted using the κ-CAM bioinks after performing Live/Dead staining with ethidium homodimer 1 (red, dead cells) and calcein AM (green, live cells), at day 8. **B** Percentage of viable cells calculated from fluorescence microscope images (*n* = 3). Statistical significance was assessed using ordinary one-way ANOVA and Tukey’s multiple comparison test (ns *P* value ≥ 0.05; * = 0.01 < *P* value < 0.05; ** = 0.0016; *** = 0.0009). The scale bar indicates 150 µm.

3D bioprinting was performed using the previously defined printing parameters. For this, an embryonic-like fish cell line derived from European sea bass (*Dicentrarchus labrax* Embryonic Cell Line, DLEC) was used, and DLEC cells were incorporated into the κ-CAM bioinks and 3D bioprinted. The viability of the cells was assessed after culturing those structures for 8 days. Figure 3A shows the fluorescence microscope images after live/dead staining of DLEC cells bioprinted in 85κ-c07AA, 72κ-c40MC at 30 °C and in 40κ-c20MC30AA, 56κ-c20MC10AA and 44κ-c25MC80AA at room temperature (RT). The highest cell viability obtained was for the cells in the 44κ-c25MC80AA bioink, corresponding to 95%. This result is most likely due to its higher alginate concentration in comparison to the other bioinks. Following that, an 89% cell viability was found for the 72κ-c40MC bioink, whereas an 88% cell viability was found for the 90κ, 85κ-c07AA and 56κ-c20MC10AA bioinks. Lastly, a cell viability of 87% was obtained for 72κ-c40MC bioink (Fig. 3B). DLEC cell viability was also evaluated after encapsulation prior to bioprinting in the different κ-CAM bioinks, showing slightly higher values in cell viability as expected (Fig. S2).

### 3D printing and printability assessment of the microalgae-enriched fat-based inks (mFAT)

**Fig. 4:**
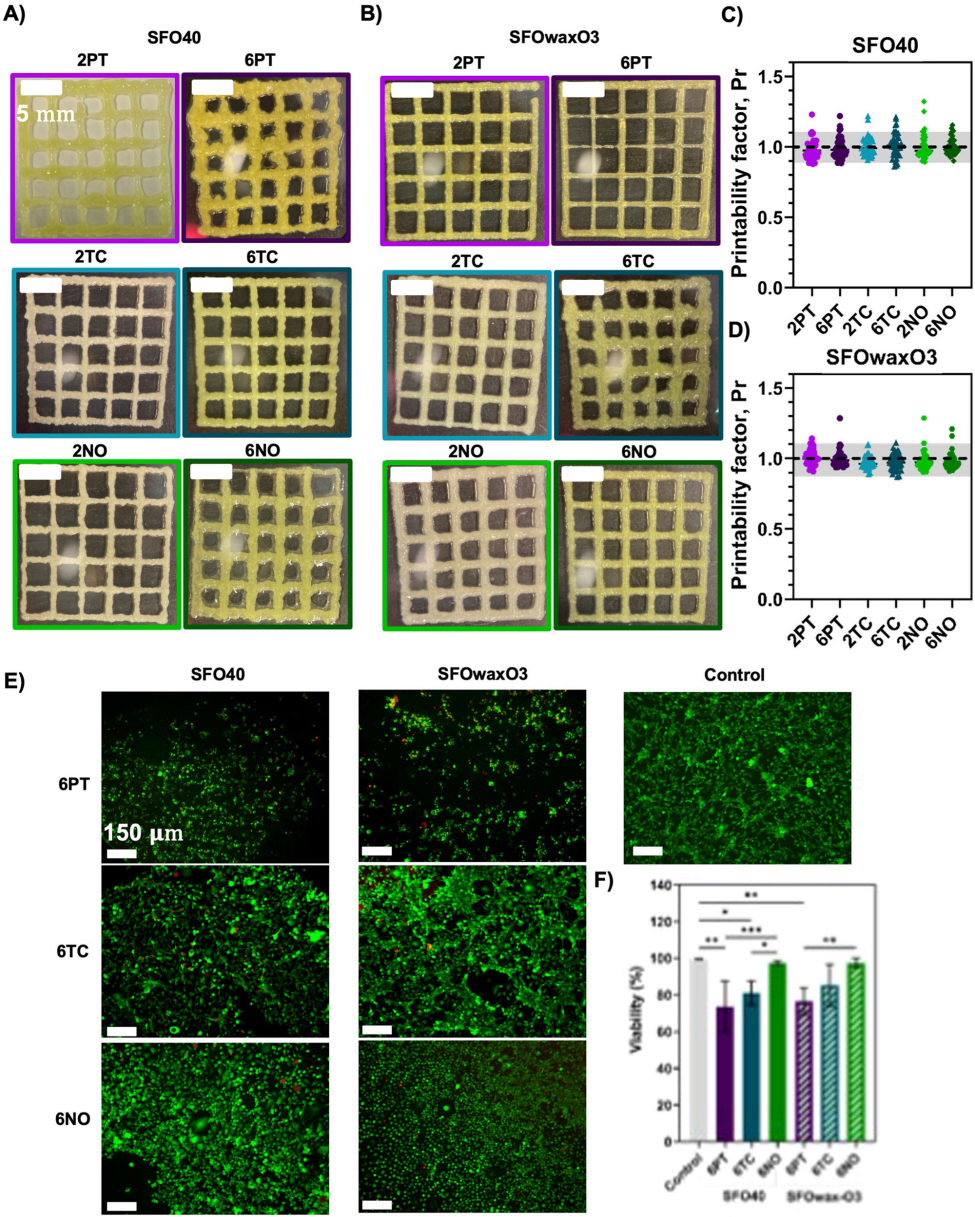
Printability and biocompatibility assessment of mFAT inks. Optical microscopy images of 3D-printed squared meshes using the fat inks **A** SFO40 and **B** SFOwaxO3 with PT, TC, and NO. Calculation of the Printability factor of the fat inks **C** SFO40 and **D** SFOwaxO3 with PT, TC, and NO with each individual data point representing the printability factor of one pore. The gray region highlights the range of adequate ink printability (from 0.9 to 1.1) as described in Ouyang et al.^40^. Data are shown as mean ± SD (*n* = 3). Scale bar: 5 mm. **E** Fluorescence microscope of DLEC cells seeded and cultured for 8 days on the top of 3D-printed fat scaffolds using the fat inks SFO40 with 6PT, 6TC, and 6NO and SFOwaxO3 with 6PT, 6TC, and 6NO after a Live/Dead staining with ethidium homodimer 1 (dead cells) and calcein AM (viable cells). Control corresponds to DLEC cells growing on a polystyrene culture plate. Scale bar: 150 μm. **F** Percentage of viable cells and estimated from the fluorescence microscope images (*n* = 3). Statistical significance was assessed using ordinary one-way ANOVA and Tukey’s multiple comparison test (ns *P* value ≥ 0.05; * = 0.01 < *P* value < 0.05; ** = 0.0016; *** = 0.0009).

To assess the impact on the organoleptic properties of inks after the addition of microalgae, several inks were also formulated and termed mFAT inks. The composition and nomenclature of these can be seen in “Methods”. Figure 4A shows the resulting 3D-printed meshes of the SFO40 inks supplemented with the different microalgae at concentrations of 0.6% or 0.2% (SFO40.6PT, SFO40.2PT, SFO40.6TC, SFO40.2TC, SFO40.6NO, and SFO40.2NO), whereas Fig. 4B shows 3D-printed meshes of SFOwaxO3 inks supplemented with 0.6% and 0.2% microalgae (SFOwaxO3.6PT, SFOwaxO3.2PT, SFOwaxO3.6TC, SFOwaxO3.2TC, SFOwaxO3.6NO, and SFOwaxO3.2NO). The printability evaluation of each set of conditions is represented in Fig. 4C, D, respectively.

### Assessment of the cytocompatibility of mFAT inks toward (bio)printing of hybrid products

To assess the cytocompatibility of the mFAT inks, DLEC cells were seeded onto the surface of 3D-printed scaffolds manufactured with the mFAT inks. For this experiment, only inks with the highest microalgae concentration were selected, as this concentration could be more detrimental to fish cell viability. Figure 4E shows the viable cells (green) and dead cells (red) on each condition after 8 days of culture. The percentages of viability were calculated from the fluorescence microscopy images are shown in Fig. 4F.

From the three microalgae evaluated, mFAT inks that contained NO (SFO40.6NO and SFOwaxO3.6NO) yielded the highest viability values, comparable to the controls, corresponding to 97.44% and 97.26%, respectively. In SFO40.6TC and SFOwaxO3.6TC the viabilities slightly decreased to 81.05% and 85.35%, respectively. SFO40.6PT and SFOwaxO3.6PT resulted in the lowest of the viabilities, corresponding to 73.70% and 76.50%, respectively.

### Preliminary characterization of the mFAT organoleptic properties

**Fig. 5:**
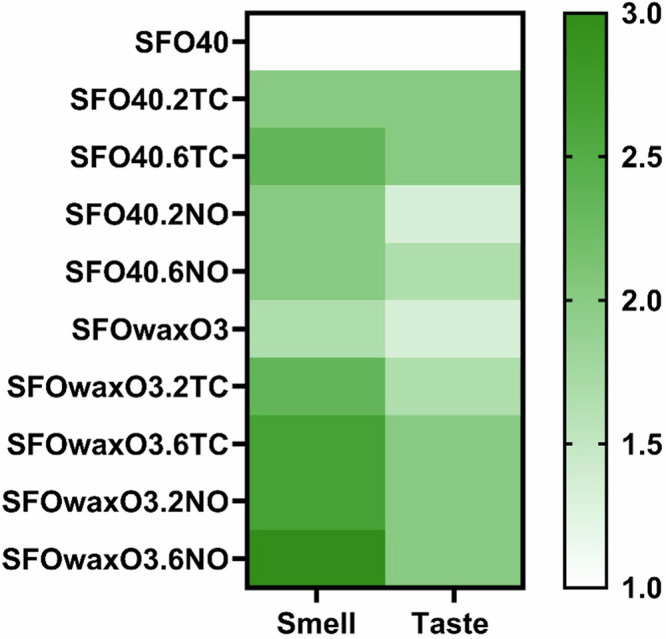
Preliminary evaluation of the impact microalgae addition on inks smell and taste. Heatmap that represents a preliminary evaluation of the impact of adding 2TC, 6TC, 2NO, and 6NO on the smell and taste of SFO40 and SFOwaxO3 hydrogels. The evaluation criteria was defined as: (1) tastes/smells like oil; (2) tastes/smells more like oil than alga; (3) tastes/smells like oil as much as alga; (4) tastes/smells more like alga than oil; and (5) tastes/smells like alga.

Preliminary characterization of the organoleptic properties of plant-based fat products with and without TC and NO can be seen in Fig. 5, where both the addition of microalgae and omega-3 oils seem to contribute towards improving the taste and smell of mFAT inks. The impact of adding such microalgae on the color of the mFAT inks through their respective RGB color code is also presented in Table S2.

### 3D printing of a plant-based calamari prototype with enhanced organoleptic properties

**Fig. 6:**
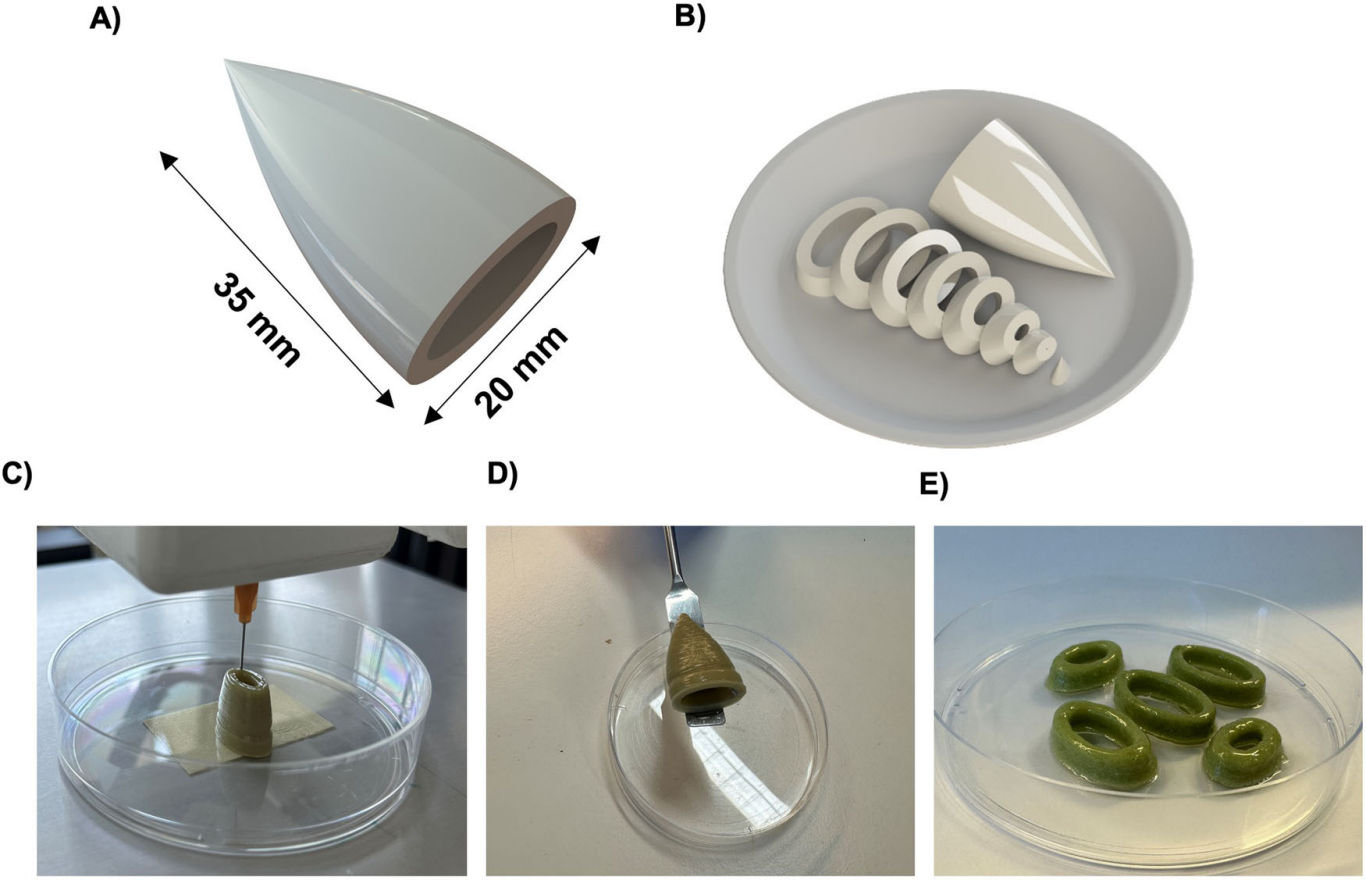
Proof-of-concept printing using mFAT inks. **A** Schematic model of an entire calamari with 35 mm of height and 20 mm wide, and **B** the respective model of the calamari in slices. **C** The 3D printing process of printing layer-by-layer the 35 mm of height calamari using the SFOwaxO3.6NO mFAT ink; **D** the 3D calamari after printed, and **E** the 3D-printed calamari slices, with O-ring shapes, using the SFO40.6NO mFAT ink.

A schematic model of a calamari (Fig. 6A) and a design of sliced calamari, shaped as O-rings (Fig. 6B), were fabricated to showcase the versatility and potential use of 3D (bio)printing in the development of cultured fish products. The ink SFOwaxO3.6NO was selected as it presented the highest smell score in the preliminary organoleptic evaluation. Figure 6C illustrates the printing process—which can also be seen in Videos S1 and S2—of this mFAT ink with high fidelity as well as robustness upon its manipulation after printing, using a spatula (Fig. 6D). Figure 6E shows that this mFAT ink can maintain structural integrity after producing a structure with 35 mm in height.

### 3D (bio)printing of a hybrid cultivated seabass product

**Fig. 7:**
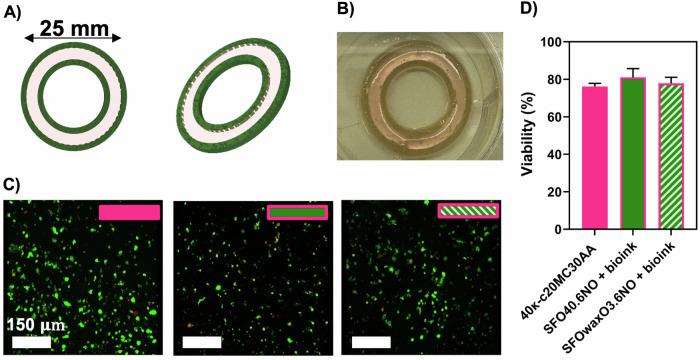
Bioprinting of a hybrid model containing both κ-CAM bioinks and mFAT inks. **A** 3D design of a slice of the 3D calamari for dual-extrusion (bio)printing—with three layers. The outer and inner internal part of the construct are made with a mFAT ink, SFO40.6NO or SFOwaxO3.6NO, and the middle ring is made with the DLEC loaded 40κ-c20MC30AA formulation (bioink). Image produced on SolidWorks. **B** 3D (bio)printed cultivated fish calamari. **C** Fluorescence confocal microscope images of DLEC cells in O-ring structures a single extruded O-ring structures made from a cell loaded 40κ-c20MC30AA bioink, (control), and three-layer dual-extruded O-rings with a middle layer made of a cell-loaded 40κ-c20MC30AA bioink and the external inner and outer layers made of a mFAT ink, either SFO40.6NO or SFOwaxO3.6NO. The scale bar indicates 150 µm. **D** Percentage of viable cells calculated from fluorescence microscope images (*n* = 3). Statistical significance was assessed using ordinary one-way ANOVA and Tukey’s multiple comparison test (ns *P* value ≥ 0.05; * = 0.01 < *P* value < 0.05; ** = 0.0016).

The 3D design of the 3D (bio)printed cultured seafood product is shown in Fig. 7A. The (bio)printing was performed using the 40κ-c20MC30AA bioink and either the SFO40.6NO or the SFOwaxO3.6NO inks. The (bio)printed structure (Fig. 7B) was maintained in culture for 15 days to allow for cell proliferation and growth within the bioink. After that, we performed a live/dead staining.

Figure 7C shows the representative fluorescent microscopic images for each condition. The cell viability within the construct was estimated (Fig. 7D) reaching cell viability values of 76.14% (control), 81.10% (SFO40.6NO), and 77.87% (SFOwaxO3.6NO), with no significant statistical differences. These are considered high viability values, specifically after 15 days of culture. We also observed that the majority of dead cells were located within the structure instead of being present at the surface (Videos S3–S5). It is to be noted that dead cells within such constructs will also remain encapsulated and will not be washed away during routine cell culture media changes.

## Discussion

When formulating bioinks for fish culture applications it is important to consider that, ideally, the materials used should be edible, able to support cell adherence and maturation, have scalability capacity and originate from non-animal sources^26^. Furthermore, assessment of the rheological properties is also key when formulating bioinks as they will provide valuable information on their behavior during the printing and post-printing processes. High shear rates are associated with the pressure applied in the nozzle during printing^27^. We demonstrated that κ-CAM bioinks have the ability to flow through the printing nozzle due to a decrease in viscosity caused by the pressure applied (shear-thinning properties), potentially facilitating the deposition of the bioink and contributing to high cell viability.

After extrusion, the bioinks must recover their initial viscosity to maintain the integrity of the 3D bioprinted structure. A bioink exhibiting this behavior is said to have thixotropic properties. Ideally, the bioinks would become thinner under the applied force in the nozzle, and after, when free of such force action, the viscosity would fully recover to its initial state^27^. Overall, all κ-CAM bioinks present a high degree of thixotropy, suitable to induce flow within the nozzle and with a speed of recovery suitable for bioprinting (<60 s)^28^.

As previously reported in the literature, the molecular arrangements of κ-c are temperature-dependent and shift, in a reversible manner, from random coil structures (high temperatures) to helix structures (low temperatures)^25,29^. This allows for the bioprinting of self-supporting structures that do not require additional supporting materials. In order to assess this temperature-dependent molecular arrangement, the viscosity of the bioinks was evaluated throughout a range of temperatures (Fig. 1C). After this evaluation, we established the working printing temperature interval for 90κ-c and 85κ-c07AA as 28–30 °C, while for 72κ-c40MC and 56κ-c20MC10AA this was established at 25–28 °C. In the case of 40κ-c20MC30AA and 44κ-c25MC80AA, there was a quicker (72 s) and less pronounced transition occurring between 23.28 and 19.89 °C. We hypothesize that this is due to their lower κ-c concentration, as κ-c exhibits the most pronounced temperature-dependent behavior among the materials used. For this reason, the working printing temperature for 40κ-c20MC30AA and 44κ-c25MC80AA was set at 20–25 °C. Overall, we consider that the κ-CAM bioinks are compatible with fish cell culture temperature requirements, typically ranging from 21 to 28 °C. This feature is crucial to maintain cell viability during the bioprinting process.

The mechanical properties of bioinks also play a predominant role in the texture of cultured fish products^23^. Different moduli are required to culture and grow different cell types. For example, moduli within 1–10 kPa are suitable for adipose tissue cells, while it is required modulus ≥10 kPa for muscle tissue cells^30–32^. More recently, Lee et al. also highlighted the importance of scaffold stiffness to enhance cell differentiation and the sensorial output of cultured meat^33^. Since the κ-CAM bioinks are within these values, we suggest that they are potential candidates for promoting adipogenesis or myogenesis and present adequate texture for cultured fish tissue development, depending on the chosen bioink. Besides being able to form robust gels with adequate mechanical strength, it is also important that these materials have a high water content, a key feature of hydrogels and important to support cell survival within the bioink matrix. All scaffolds showed high water contents, which has been reported in the literature as adequate to support high cell viabilities^25^.

Before proceeding to the bioprinting of DLEC cells with the κ-CAM bioinks, a preliminary assessment of the printability was performed. All κ-CAM were considered to have adequate printability. Therefore, these bioinks can be used to fabricate 3D structures with adequate fidelity. However, we observed printability differences between bioinks. For example, the 3D structure printed using 90κ-c presents a lower resolution than the 3D structure printed using 56κ-c20MC10AA. This was also visible in the printability evaluation with 90κ-c presenting a high dispersion of values within the 0.9–1.1 window, while most of the printability values of 56κ-c20MC10AA are very close to 1. We hypothesize that this result could be related to the bioink composition. Still, the 40κ-c20MC30AA, 56κ-c20MC10AA and 44κ-c25MC80AA are printable at temperature ranges closer to the ones suitable for fish cell culture^4^.

We also assume that DLEC cells will contribute to the nutritional value of the final printed product by providing animal protein. For this, it is therefore required that cells are able to grow and rearrange inside the material and cells viabilities were measured post-printing. The cell viability was above 80% in all cases, being higher in the 44κ-c25MC80AA bioink. This result is most likely due to its higher alginate concentration in comparison to the other bioinks. The presence of dead cells was negligible in all bioinks, therefore, we can conclude that the hydrogels are cytocompatible with DLEC cells and non-toxic. Furthermore, we showed that the 3D bioprinting process does not significantly affect viability when compared to DLEC cell encapsulation. Moreover, all cell viabilities obtained using 3D bioprinting fall within the range of the values reported by other studies on extrusion-based bioprinting. Interestingly, the bioprinting at 30 °C did not result in lower cell viability compared to RT. We hypothesize that this is due to the short duration of the bioprinting process. Furthermore, these bioinks could also be good candidates to perform 3D bioprinting of other food-relevant animal cells that grow at higher temperatures, such as avian, mammalian, or Atlantic mackerel cells, which are cultivated at 28 °C^34,35^.

In the context of cultured meat/fish production, maintaining cell viability within the final construct, may attend different purposes and strategies, such as: (i) Decreasing the need of higher number of cells in the bioink formulations by starting with low cell concentrations to facilitate cell growth within the scaffold over time. To support this process, incorporating focal adhesion points into the bioink formulation would be advantageous, as it promotes cell attachment, expansion, and, eventually, cues for early cell culture fate. While cell attaching to macroalgae-based materials is challenging, we have previously demonstrated that bioinks, composed solely of ĸ-c, can successfully support cell proliferation and elongation^25^. (ii) Alternatively, for strategies that rely on seeding a high number of cells, cell adhesion to scaffolds and/or formation of cell-to-cell adherent constructs have the potential to improve the texture of the final product and enhance its organoleptic properties, such as taste, aroma, and mouthfeel. In this regard, the mechanical properties of the final product are expected to evolve over time as the tissue matures and integrates. To fully evaluate these two strategies—low cell density with scaffold-facilitated growth versus high cell density for immediate texture optimization—long-term experiments would be required. These experiments fall outside the scope of this paper, which focuses on the formulation and characterization of bioinks, as well as 3D (bio)printing to demonstrate biocompatibility and structural features.

The use of 3D printable vegan fat-based inks in the cellular agriculture field was first proposed by Schüler et al.^36^. Here, we initially evaluated the impacts of adding different microalgae with reported sea-like odor and flavor to inks and their effects on 3D printing process and printability. We hypothesize that the combination of the mFAT inks with the κ-CAM bioinks during the 3D (bio)printing process can be advantageous for structure integrity, sensorial properties, and nutritional value of cultured fish products. For this, we evaluated the addition of three microalgae reported as sensorial enhancers in fish products: NO, TC, and PT^15,37,38^. mFAT inks were developed based on previous work, with their characterization described in detail elsewhere^36^. In general, the addition of microalgae resulted in printability values very close to 1, being considered adequate to perform 3D printing. However, we observed that inks with higher microalgae concentrations, such as the inks SFO40.6PT, SFO40.6N, and SFOwaxO3.6TC, led to the 3D-printed structures being more deformed than their counterpart inks with lower microalgae concentrations. In addition, it seems that overall, the addition of carnauba wax contributes to maintaining an adequate printability when increasing the microalgae concentration, as shown for SFOwaxO3.6PT and SFOwaxO3.6NO.

The combination of different inks during the 3D (bio)printing process for the development of hybrid cellular agriculture products should also allow the survival of the cells^39^. Therefore, even though the mFAT inks are not formulated to contain cells, they are in direct contact with the κ-CAM bioinks, which will host the DLEC cells. In this sense, the cytocompatibility of mFAT inks and DLEC was assessed. From the three microalgae evaluated, mFAT inks that contained NO (SFO40.6NO) and SFOwaxO3.6NO yielded the highest viability values, while SFO40.6TC and SFOwaxO3.6TC showed slightly lower viability. SFO40.6PT and SFOwaxO3.06PT resulted in the lowest viability. We hypothesize that this decrease in cell viability could be related to the culture conditions of those microalgae and the processes to collect and purify the biomass. From this study, we concluded that the addition of NO and TC biomass maintains high DLEC viability, while the addition of PT biomass was considered slightly cytotoxic. In addition, we observed differences in DLEC morphology in the presence of the different microalgae biomass, as DLEC cells cultured in the presence of SFO40.6TC and SFOwaxO3.6TC were elongated, while DLEC cells cultured on SFO40.6NO and SFOwaxO3.6NO were rounder. Still, this morphological behavior did not affect cell viability, thus for further preliminary evaluation of the organoleptic properties and to perform 3D (bio)printing using microalgae-containing inks, the microalgae NO and TC were selected.

Besides the aforementioned sensorial properties of the three microalgae selected in this study, they are high producers of EPA and DHA, which are considered vital omega-3 in human diet^40^. Moreover, Coleman et al. demonstrated that these microalgae are promising flavoring agents and odor enhancers for alternative seafood products^15^. Based on this, we anticipated that the addition of microalgae could be important to improve the sea-like odor and taste of cultured fish products. In order to demonstrate that, we performed a preliminary assessment, with a human panel, of the impact on the smell and flavor of the mFAT inks. Regarding the taste, the panel considered that the fattiness was maintained, being a main flavor component; still in the presence of the microalgae the panel pointed out that the product tasted more like oil than algae (score 2), rather than just like oil (score 1). The same was observed regarding the smell. The addition of NO to SFOwaxO3 (SFOwaxO3.6NO) resulted in the highest scored value, with the panel selecting a score 3 for this material, indicating that this product smells like oil as much as algae; unlike the fat-based ink SFO40 which scored only 2. We hypothesize that this may be due to the presence of omega-3 (O3) oil in the formulation of the SFOwaxO3 inks. In fact, odor-active volatile components such as fatty acids-derived compounds were identified as important seafood aroma compounds in microalgae^15^.

Although this was a preliminary assessment, we aim with these results to illustrate the importance of tuning the organoleptic properties while formulating novel food products, as it can improve human perception and acceptance of those. Here, we confirmed that the addition of specific microalgae (NO and TC) can be crucial for enhancing the smell and taste of alternative fish products.

Besides affecting the odor and flavor of the fat inks, the addition of microalgae also impacts the color of the final product. When processing and cooking food, the visual appearance is highly relevant to enhance consumer acceptance of the product^41^. For that, we determined the color of the microalgae-containing fat inks through their respective RGB color code (see Supplementary data Table S2). While the plain SFO40 and SFOwaxO3 inks were considered beige, their microalgae-infused counterparts were considered dark green, dry green, and dark yellow. These colors are present in several food products but are not commonly associated with fish products. Therefore, we conclude that microalgae-containing inks are not suitable for all types of seafood consumers. Nevertheless, there are several compatible food products as breaded fish fillet, calamari and fish fingers, where the inks could be hidden inside the product and their contribution to the visual appearance is diminished or used for specific marketing purposes. On the other hand, with creative designing ideas for 3D (bio)printing it could be possible to create specific printing patterns that do not expose these inks colors to the surface of the final printing product, allowing them to still contribute to the organoleptic properties.

The mFAT ink SFO40.6NO was also selected for the subsequent studies as it presents the highest cytocompatibility with DLEC cells (97.44% DLEC viability), and it was able to maintain the structural integrity of a 3D printed calamari with 35 mm in height. This showcases the ability of the novel microalgae-containing inks to produce complex structures. Therefore, SFO40.6NO could potentially be used to produce hybrid 3D (bio)printed cultivated seafood products with enhanced organoleptic properties.

In order to prototype a 3D (bio)printed hybrid product, we dual-extruded an mFAT ink with the bioink containing the DLEC cells, combining them in the same construct. DLEC cells grown in this hybrid structure demonstrated high cell viability after 15 days of culture. From this study, we confirmed the compatibility of both mFAT inks and κ-CAM bioinks to prototype complex seafood products with enhanced taste and smell and their potential to be exploited in the manufacturing of hybrid cultured fish products.

In conclusion, we demonstrate in this work an algae- and plant-based multi-material 3D (bio)printing process for the fabrication of a cultured seafood product with enhanced organoleptic properties. Initially, the κ-CAM bioinks’ mechanical and rheological properties were assessed. The rheological properties allowed us to predict the bioprinting process since we were able to understand the effect of temperature on the gelation of the material and kinetics, while the mechanical properties confirmed that we were working within Young’s modulus values suitable for the development of fat products. 3D-printed squared meshes with such bioinks were fabricated with adequate printability. DLEC embryonic-like seabass cells were incorporated into the bioinks formulation, maintaining high viability after the bioprinting process. Besides this, several microalgae reported as enhancers of sea-like organoleptic properties were used in the formulation of mFAT inks that also showed adequate printability. A preliminary assessment of the microalgae-containing mFAT inks demonstrated their potential for enhancing the smell and flavor of the final product. A cultured 3D (bio)printed calamari with DLEC cells incorporated was fabricated to showcase the potential of combining both κ-CAM bioinks and mFAT inks. Overall, this study introduces a novel approach to produce hybrid 3D (bio)printed cultured seafood products from plant and algae-based materials.

## Methods

### κ-CAM bioinks formulation

κ-c and MC were purchased from Sigma Aldrich (St. Louis, MO, USA). AA sodium salt, low viscosity, was purchased from MP Biomedicals (Fountain Parkway, Solon, USA). The solvent consisted of Leibovitz 15 aqueous medium (Sigma Aldrich) containing 1% (v/v) l-Glutamine (Sigma Aldrich) supplemented with 5 µL mL^−1^ of 3 M NaCl, named subsequently as bioprinting media. κ-c solutions, at concentrations of 0.9% (w/v) and 0.8% (w/v,) were prepared by mixing κ-c in bioprinting media under continuous stirring in a hotplate (Stuart, Cole-Palmer, Vernon Hills, IL, USA) at 80 °C until fully dissolved. 2% (w/v) and 1% (w/v) MC solutions were prepared by mixing MC in bioprinting media under continuous stirring at room temperature (RT) until fully dissolved. Afterward, both solutions were kept at 4 °C overnight. AA solutions, at concentrations of 4% (w/v), 1.5% (w/v) and 1% (w/v), were also prepared by mixing AA in bioprinting media under continuous stirring at RT until fully dissolved. κ-CAM bioinks were formulated by combining the κ-c, MC, and AA solutions at different ratios, as described in Table 1.

**Table 1:** Concentrations and ratios of the components of the κ-CAM bioinks and respective nomenclature

Nomenclature	Solute concentration in initial solutions (% w/v)	Ratios of solutions used for formulation of the κ-CAM bioinks (κ-c:MC:AA)	Final concentration in the κ-CAM bioinks (% w/v)
90κ-c	κ-c 0.9%	1:0:0	κ-c 0.9%
85κ-c07AA	κ-c 0.9%, AA 1.5%	9.5:0:0.5	κ-c 0.855%, AA 0.075%
72κ-c40MC	κ-c 0.9%, MC 2%	8:2:0	κ-c 0.72%, MC 0.4%
40κ-c20MC30AA	κ-c 0.8%, MC 1%, AA 1%	5:2:3	κ-c 0.40%, MC 0.2%, AA 0.3%
56κ-c20MC10AA	κ-c 0.8%, MC 1%, AA 1%	7:2:1	κ-c 0.56%, MC 0.2%, AA 0.1%
44κ-c25MC80AA	κ-c 0.8%, MC 1%, AA 4%	5.5:2.5:2	κ-c 0.44%, MC 0.25%, AA 0.8%

*κ-c* κ-carrageenan, *MC* methylcellulose, *AA* alginic acid sodium salt.

### mFAT inks formulation

mFAT inks were formulated by adapting a previously described protocol by Schüler et al.^36^. First, a 2% (w/w) soy protein isolate (SPI) suspension in ultrapure water was prepared, magnetically agitated at 50 °C for 5 h, and left overnight at 6 °C. Ultrapure water was then added to reach a final 1% (w/w) SPI concentration. Such solution was pre-heated at 50 °C under magnetic stirring to prepare two different emulsions: a mFAT ink (SFO40), consisting of 40% (w/w) of sunflower oil (SFO) and 2% (w/w) of ĸ-c; and mFAT ink (SFOwaxO3) consisting of 90% (w/w) SFO, 20% (w/w) carnauba wax (Cwax), and 5% (w/w) algae oil (O3). Both mFAT inks were prepared by adding the components to the previously formulated SPI suspension and homogenizing the resulting mixture using a T10 basic Ultra-Turrax (IKA). Subsequently, 1.5% (w/w) κ-c was added to this mixture and homogenized in an T10 basic Ultra-Turrax (IKA).

NO, TC, and PT biomass powder were purchased from Algikey–Algae Based Solutions S-A (Póvoa de Santa Iria, Portugal). SFO40 and SFOwaxO3 mFAT inks were first heated to 80 °C using a magnetic stirring hot plate. The microalgae biomass powder was then added at either 0.2% (w/v) or 0.6% (w/v) concentrations, and the mixtures were homogenized using a T10 basic Ultra-Turrax. The nomenclature used for each type of mFAT ink in this study is provided in Table 2.

**Table 2:** Concentrations and ratios of the components of the mFAT inks and respective nomenclature

Nomenclature	Plant-based fat component (% w/w)	Microalgae biomass (% w/v)
SFO40	SFO 40%	–
SFO40.6PT		PT 0.6%
SFO40.2PT		PT 0.2%
SFO40.6TC		TC 0.6%
SFO40.2TC		TC 0.2%
SFO40.6NO		NO 0.6%
SFO40.2NO		NO 0.2%
SFOwaxO3	Cwax 1%, SFO 18%, O3 1%	–
SFOwaxO3.6PT		PT 0.6%
SFOwaxO3.2PT		PT 0.2%
SFOwaxO3.6TC		TC 0.6%
SFOwaxO3.2TC		TC 0.2%
SFOwaxO3.6NO		NO 0.6%
SFOwaxO3.2NO		NO 0.2%

*SFO* sunflower oil, *Cwax* carnauba wax, *O3* algae oil, *PT* *Phaeodactylum tricornutum*, *TC* *Tetraselmis chuii*, *NO* *Nannochloropsis oceanica*.

### Cell culture

DLEC cells were purchased from Kerafast (Boston, MA, USA) and cultured as described by Buonocore et al.^42^. Briefly, culture media (complete L-15 media) was prepared using Leibovitz 15 medium (L-15 medium, Sigma Aldrich) supplemented with 10% (v/v) fetal bovine serum (FBS, Thermo Fisher) 1% (v/v) l-glutamine (Thermo Fisher), 1% (v/v) antibiotic–antimycotic solution (Thermo Fisher) and 5 µL mL^−1^ of 3 M NaCl. DLEC cells were incubated at 25 °C and media was changed every 2–3 days. Cell passaging was performed every 4–5 days using 0.05% trypsin/ethylenediaminetetraacetic acid (EDTA, Thermo Fisher) for 8 min at RT. Cells were recovered by centrifugation at 160 G for 4 min. After carefully removing the supernatant, the cell pellet was resuspended in L-15 complete media. Cells were seeded at a density of 10 000 cells cm^–2^ into new plates containing complete L-15 media.

### Preliminary 3D (bio)printing optimization

Preliminary optimization of the (bio)printing parameters and printability evaluation was carried out using a custom-made microextrusion-based 3D printing system, as described previously^43^. This system consists of a three-axis dispensing robot Fisnar F4200N.2, (FISNAR, Germantown, WI, USA) connected to a pneumatic dispensing unit (DC100 High Precision Dispenser, Ellsworth, Glasgow, UK) and interfaced with a personal computer. Syringe barrels, barrel adaptors, end caps, pistons, and dispensing tips were also provided by Ellsworth. Digital models were uploaded using the Fisnar RobotEdit software (FISNAR, Germantown, WI, USA). Parameters including printing speed, applied pressure, and distance between layers were modified to determine the optimal printing conditions.

For the (bio)printing of complex 3D structures, an in-house built bioprinter adapted from a 3D Fused Deposit Modelling (FDM) printer (Creality, Ender-2 V2) was used^44^. Computer-Aided Design (CAD) models were designed on SolidWorks (Dassault Systèmes, SolidWorks Corp., France) and exported as .stl files, modified in Cura (Ultimaker Cura, Netherlands) and executed in Pronterface (Printrun, the Netherlands).

### Bioprinting of the κ-CAM bioinks

A 3-mL syringe barrel and 23-gauge dispensing tips were used. The 90κ-c bioink was printed at 37 °C; the 85κ-c07AA and 72κ-c40MC bioinks were printed at 30 °C; and the 40κ-c20MC30AA, 56κ-c20MC10AA and 44κ-c25MC80AA bioinks were printed at 20 °C. All materials were printed at a print speed of 10 mm s^−1^. Printing pressure varied between 4 and 14.5 psi.

When bioprinting, cells were incorporated into the κ-CAM bioinks. For this, cells were suspended and centrifuged following the procedure described in the “Cell culture” section. The cell pellet was then resuspended in 250 µL of L-15 complete media. Afterward, 1,000,000 cells mL^−1^ were pipetted into the κ-CAM bioink. To ensure homogenization, the mixture was pipetted up and down thoroughly.

### 3D printing of mFAT ink

3-mL syringe barrels and 23-gauge dispensing tips were also used in the printing of mFAT inks. The inks were printed at 40 °C at a printing speed of 10 mm s^−1^. Printing pressure varied between 15 and 22 psi.

### Printability evaluation

The printability of the κ-CAM bioinks and mFAT inks was quantified by their ability to form square-shaped pores. For this, 20 × 20 mm meshes were 3D-printed, each containing 25 pores.

As previously reported^25,39^, the printability factor (Pr) was calculated based on the ratio of the theoretical (C’) to the experimental (C) circularity. For a square shape, C′ is equal to π/4. Therefore, Pr was measured considering the pore perimeter (L) and the pore area (A) using Eq. (1):

1 $$\Pr =\frac{\pi }{4}\times \frac{1}{C}=\frac{{L}^{2}}{16A}$$

Where, an ideal gelated ink with square-shaped pores corresponds to Pr = 1, Pr < 1 corresponds to under-gelated inks and rounded pore corners, and Pr > 1 to over-gelated inks.

### Evaluation of mechanical properties

First, hydrogels were produced by casting the bioink solutions into cylindrical molds (4–6 mm of diameter and 4 mm of height, *n* = 5), and kept at 6 °C overnight. Next, the samples were incubated with 2 mL of complete L-15 medium and 0.5 M KCl (100 µL mL^−1^) and placed in a incubator at 25 °C for 30 min to mimic the post-bioprinting culture conditions.

A Univert (CellScale Biomaterials Testing) load frame with a 10 N load cell was used to conduct a uniaxial compression test at a constant displacement rate of 3 mm min^−1^. Stress–strain curves were plotted, as previously described^25^. Young’s modulus was estimated using the 0–15% linear strain section of the stress–strain curve (Eq. (2)). The mechanical properties were evaluated on the κ-CAM bioinks.

2 $${Youn}{g}^{{\prime} }{s\; modulus}=\frac{\sigma }{\varepsilon }$$

Where σ (Nmm^−2^) corresponds to stress and ε (non-dimensional) to strain.

### Rheological assessment

The rheological properties of the κ-CAM bioinks were assessed using an MCR 92 modular compact rheometer (Anton Paar). A cone-plate geometry was used for the rheological analysis in the viscoelastic zone, with a cone diameter of 50 mm, a constant measurement gap of 0.1 mm, and a sample volume of 0.5 mL. The shear-thinning properties of the bioinks were determined by following the viscosity as the shear rate increased. Rotational recovery measurements were also performed to characterize the bioink recovery behavior by applying an initial shear stress of 2 Pa for 60 s, followed by 15 Pa during 30 s, and a final shear stress of 2 Pa for 60 s. Moreover, the elastic (G’) and viscous (G”) modulus were measured over 360 s during a temperature sweep from 37 °C to 20 °C, with a rate of 0.2 °C s^−1^. All experiments were performed at 25 °C, except for 90κ-c and 85κ-c07AA bioinks, whose measurements were performed at 37 °C and 30 °C, respectively. Each bioink measurement was performed in triplicate (*n* = 3).

### Cell seeding onto 3D-printed mFAT scaffolds

Cell seeding was performed on top of 10 × 10 mm square meshes, 3D-printed with the mFAT inks, to evaluate their compatibility with DLECs. These structures were printed with three layers in height in 12-well plates at 40 °C under sterile conditions. The scaffolds were sterilized with 2% (v/v) antibiotic/antimycotic solution in PBS overnight at 4 °C, followed by UV sterilization for 10 min. 100 000 DLEC cells were seeded on each sample. Structures were initially incubated with a low volume of complete L-15 for 2 h to promote initial cell attachment. Afterward, complete L-15 was added covering the whole scaffold. Controls consisted of 100,000 DLECs cultured in the absence of the printed structures. DLECs were cultured for 8 days at 25 °C and the culture media was changed every 2–3 days.

### Cell viability assay

A Live/Dead assay was performed to evaluate the viability of the DLECs bioprinted using the κ-CAM bioinks and to assess the cytocompatibility of the DLECs cultured on the mFAT inks structures. For this, cells were incubated for 30 min with 1 µM acetoxymethyl (AM) calcein (Sigma Aldrich) to stain viable cells (green), while dead cells were stained with 5 µM ethidium homodimer I (Sigma Aldrich) (red). Images were obtained on a Leica DMI3000B fluorescence microscope (Leica Microsystems). Three representative images were taken for each structure (*N* = 3, *n* = 3). The resulting images were used to quantify live and dead cells using ImageJ software (ImageJ 1.51f, National Institutes of Health, Bethesda, MD, USA). For the visualization of the bioprinted cells on the hybrid structures (see “Bioprinting of a hybrid structure”), fluorescence images were taken using a Zeiss LSM980/Airyscan2 confocal microscope. The datasets were processed and reconstructed on Image J.

### Human panel evaluation of flavor and smell

Samples were fabricated with the mFAT inks by casting them using cylindrical molds (4–6 mm of diameter and 4 mm of height). Samples were sterilized under UV for 60 min. All materials used were sourced from food suppliers or with a level of purity that ensures food safety. All instruments, vessels, and molds used in the preparation of the samples were carefully cleaned and washed. The panel (*n* = 3) assessed the flavor and smell of the mFAT inks. The panel participants were comprised by individuals aged between 20 and 25 and were not trained in algae and oil flavor/odor detection. The evaluation was based on the following criteria: score 1—tastes/smells like oil sample; score 2—tastes/smells more like oil than algae sample; score 3—tastes/smells like oil as much as algae sample; score 4—tastes/smells more like algae than oil sample; score 5—tastes/smells like algae sample.

The underlying assumption on this study is that seafood flavor and smell are associated to the selected microalgae biomass.

### Computational color evaluation

For the color evaluation, structures made with the mFAT inks were manufactured by casting them using cylindrical molds (4–6 mm of diameter and 4 mm of height). The mFAT structures were then placed inside a white box to photograph them under the same illumination settings. The eyedropper tool in PowerPoint (Microsoft®) was used to obtain the RGB color codes and respective color tonalities.

### Printing of proof-of-concept complex structures with the mFAT inks

SFO40.6NO and SFOwaxO3.6NO were the mFAT inks selected for extrusion bioprinting. Two different case-study models were created: (i) a calamari with 35 mm of height and 20 mm  width; and (ii) five slices (7 mm height each), with O-ring shape, representing cuts of the previous calamari structure. The models were created using SolidWorks (Dassault Systèmes, France), and sliced with a layer height of 0.33 mm using Ultimaker Cura v5.0 (Ultimaker B.V., Utrecht, Nederland). The mFAT inks were deposited at 40 °C with a printing speed of 30 mm s^-1^ and a deposition rate of 8%.

### Bioprinting of a hybrid structure

Each hybrid structure was printed using the 40κ-c20MC30AA κ-CAM bioink and the SFO40.6NO or SFOwaxO3.6NO mFAT inks. The bioprinted model consisted of a three-layered O-Ring structure, with the middle ring made with cell-loaded κ-CAM bioink (internal diameter = 17.08 mm/external diameter = 22.36 mm) sandwiched between an inner (internal diameter = 14.44 mm/external diameter = 17.08 mm) and an outer (internal diameter = 22.36 mm/external diameter = 25 mm) rings made of the same mFAT ink. The model height corresponded to 1.32 mm and was created using SolidWorks (Dassault Systèmes, France), and sliced with a layer height of 0.33 mm using Ultimaker Cura v5.0 (Ultimaker B.V., Utrecht, Netherlands). The 40κ-c20MC30AA bioink was bioprinted at 25 °C with a printing velocity of 15 mm s^−1^ and a deposition rate of 1–2% set on PronterFace (https://www.pronterface.com/). The mFAT inks were deposited at 40 °C with a printing speed of 30 mm s^−1^ and a deposition rate of 8%. Structures were 3D bioprinted into six-well culture plates and L-15 complete medium supplemented with 0.5 M KCl (100 μL mL^−1^) was added. The bioprinted structures were cultured for 14 days at 25 °C. The medium was changed every two to three days and a Live/Dead assay was performed on day 14.

### Statistical analysis

Data is presented as mean values ± standard deviations. Each experiment was conducted in triplicate (*n* = 3) unless stated otherwise. Statistical significance was performed through *t* Student tests using GraphPad Prism 10 (GraphPad, San Diego, CA, USA).

## Supplementary information

Edible bioinks SI vreviewed_final

VideoS1

VideoS2.mp4

VideoS3

VideoS4

VideoS5

## Data Availability

The data generated from this work is available and can be provided upon request to the corresponding authors.
