# Supplementary material for: Microalgae-enriched (bio)inks for 3D bioprinting of cultured seafood
Source: NPJ Sci Food. 2025 Feb 12;9:23. doi: 10.1038/s41538-025-00386-y (PMC11821890; doi:10.1038/s41538-025-00386-y)
Supplement: Supplementary file 1 — Edible bioinks SI vreviewed_final [file 41538_2025_386_MOESM1_ESM.pdf]

SUPPORTING INFORMATION

Microalgae-enriched bioinks for 3D bioprinting of cultured seafood

Diana M. C. Marques[1,2], Madalena Jabouille[1,2], Afonso Gusmão[1,2,3], Marco Leite[3], Paola Sanjuan-Alberte[1,2,]*, Frederico Castelo Ferreira[1,2]*

[1]Department of Bioengineering and Institute for Bioengineering and Biosciences, Instituto Superior Técnico, Universidade de Lisboa, Av. Rovisco Pais, 1049-001 Lisbon, Portugal

[2]Associate Laboratory i4HB—Institute for Health and Bioeconomy, Instituto Superior Técnico, Universidade de Lisboa, Av. Rovisco Pais, 1049-001 Lisbon, Portugal

[3]IDMEC, Instituto Superior Técnico, Universidade de Lisboa, Av. Rovisco Pais, 1049-001, Lisbon, Portugal

*Corresponding authors: frederico.ferreira@tecnico.ulisboa.pt , paola.alberte@tecnico.ulisboa.pt

Table S1. Comprehensive analysis of data obtained from the gelation kinetics assay of the κ-CAM bioinks.

Bioink	Gelation temperature range (°C –°C)	Gelation temperature interval (°C)	Duration of gelation (s)	Storage modulus after gelation (Pa)
90κ-c	29.37 – 19.94	9.43	90	783.36
85κ-c07AA	33.42 – 19.98	13.44	96	450.60
72κ-c40MC	27.66 – 19.84	7,82	120	512.50
40κ-c20MC30AA	22.05 – 19.89	2,16	72	100.89
56κ-c20MC10AA	27.66 – 20.04	7,62	72	210.59
44κ-c25MC80AA	23.28 – 19.91	3,31	72	99.81

Table S2. RGB codes and respective color name of the SFO40 and SFOwaxO3 microalgae-containing inks. The codes were generated using PowerPoint.

Ink	RGB code	Color name
SFO40	178,180,166	Beige
SFO40.6PT	69,78,11	Dark green
SFO40.2PT	128,131,76	Dry green
SFO40.6TC	77,92,34	Dark green
SFO40.2TC	113,121,79	Dry green
SFO40.6NO	70,70,2	Dark yellow
SFO40.2NO	84,83,2	Dark yellow
SFOwaxO3	175,174,153	Beige
SFOwaxO3.6PT	51, 60,3	Dark green
SFOwaxO3.2PT	109,110,40	Dark yellow
SFOwaxO3.6TC	98,108,49	Dark green
SFOwaxO3.2TC	96,106,56	Dark green
SFOwaxO3.6NO	70,73,27	Dark yellow
SFOwaxO3.2NO	98,100,55	Dry green

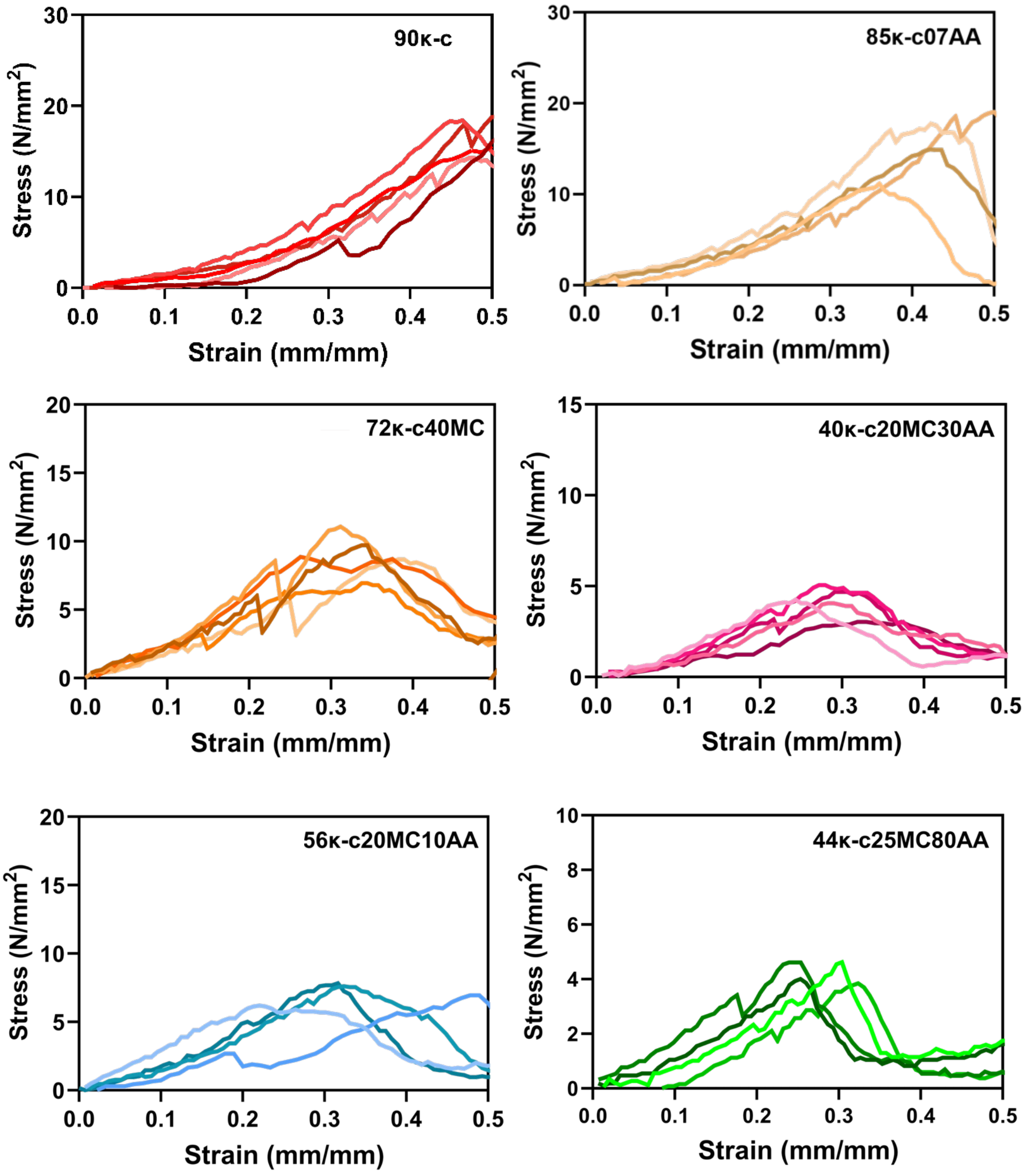

Figure S1. Stress-strain curves for the different κ-CAM bioinks.

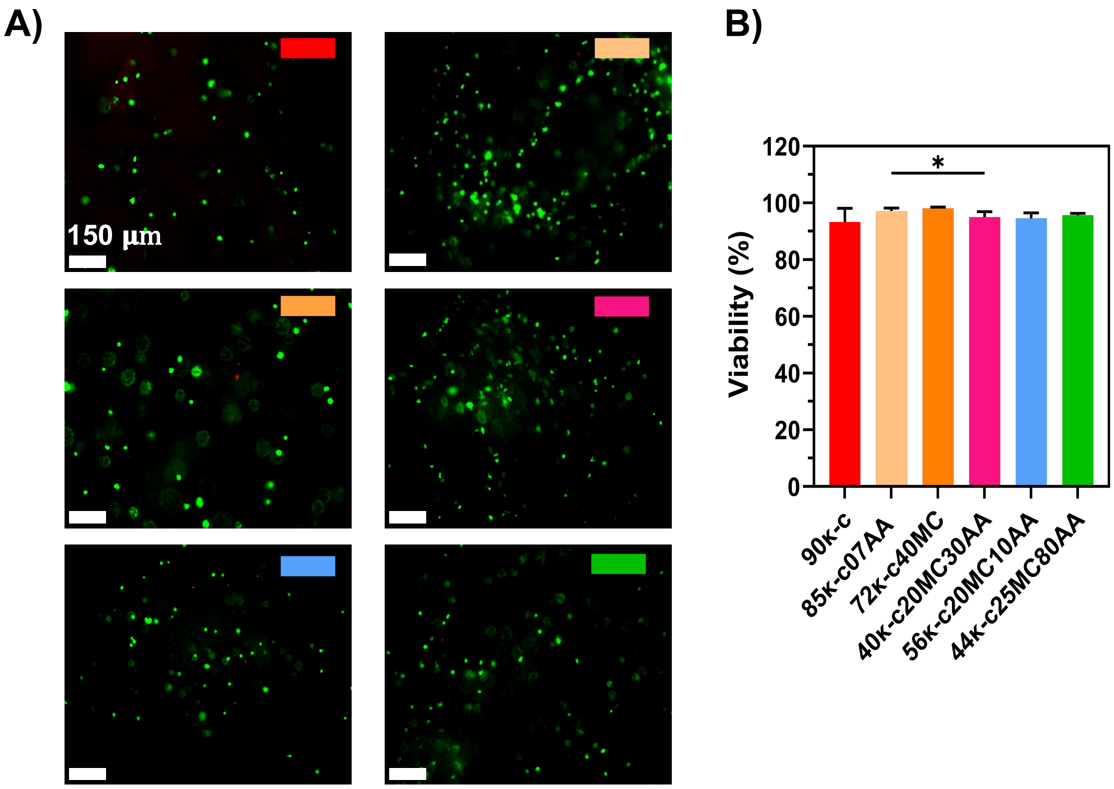

Figure S2. A) Fluorescence microscope images of DLEC cells encapsulated using the κ-CAM bioinks after performing Live/Dead staining with ethidium homodimer 1 (red, dead cells) and calcein AM (green, live cells), at day 8. **B)** Percentage of viable cells calculated from fluorescence microscope images (n=3). Statistical significance was assessed using t-student analysis (ns p value ≥ 0.05;*=0.01 < p value < 0.05; ** = 0.0016; *** = 0.0009). The scale bar indicates 150 µm. A specific color was allocated to the data for each ink, and used on both panels, as labelled in the panel B.

Video S1. 3D printing of the calamari in slices model using the SFOwaxO3.6NO mFAT ink.

Video S2. 3D printing of the complete calamari model using the SFOwaxO3.6NO mFAT ink.

Video S3. Animation of the 3D reconstruction of the confocal images taken to the 40κ-c20MC30AA bioink (control), after Live/Dead staining with ethidium homodimer 1 (dead cells) and calcein AM (viable cells), at day 15.

Video S4. Animation of the 3D reconstruction of the confocal images taken to the40κ-c20MC30AA plus SFO40.6NO, after Live/Dead staining with ethidium homodimer 1 (dead cells) and calcein AM (viable cells), at day 15.

Video S5. Animation of the 3D reconstruction of the confocal images taken to the 40κ-c20MC30AA plus SFOwaxO3.6NO after Live/Dead staining with ethidium homodimer 1 (dead cells) and calcein AM (viable cells), at day 15.
